# The Design-Driven Innovation Path of Human-Centered Artificial Intelligence in the Field of Healthcare: Theory, Practice, and Future Prospects

**DOI:** 10.3390/healthcare14142031

**Published:** 2026-07-08

**Authors:** Yuqi Liu

**Affiliations:** Department of Industrial Design, School of Design, South China University of Technology, University City Campus, Panyu District, Guangzhou 510006, China; yuqiliu@scut.edu.cn; Tel.: +86-15611129337

**Keywords:** human-centered artificial intelligence, design-driven innovation, healthcare, implementation science, human–machine collaboration, explainable artificial intelligence, ethical governance

## Abstract

**Background/Objectives**: The application of artificial intelligence (AI) in the healthcare field continues to deepen, with the development paradigm gradually shifting from technology-driven innovation to design-driven innovation towards “human-centered artificial intelligence (HCAI).” This aims to bridge the potential of AI technology with actual clinical needs and improve the quality and accessibility of healthcare services. However, it still faces challenges such as insufficient integration of theoretical frameworks, complex implementation challenges, and an imperfect ethical governance mechanism. **Methods**: This article presents a systematic narrative review about the philosophical and ethical foundations of HCAI related literature, analyzes the specific clinical application models recorded in the literature, integrates key theories related to implementation science, human–machine collaboration, and explainable AI(XAI), and constructs a multidimensional comprehensive analysis framework for HCAI in the medical field. **Results**: The study shows that design-driven innovation is the key to bridging the gap between the potential of AI technology and practical medical applications; the successful implementation of “human-centered artificial intelligence” relies on interdisciplinary collaboration, stakeholder co-creation, and ethical considerations throughout the entire lifecycle; Among them, human-centered design ensures that technology meets real needs; Implementation Science guarantees innovation can effectively integrate into complex medical environments; explainable AI technology is the cornerstone of establishing clinical trust; the strategic governance framework sets boundaries and tracks for the healthy development of the entire ecosystem. **Conclusions**: Beyond summarizing existing research findings, this study proposes targeted design frameworks and trade-off strategies for key technical and practical dilemmas of HCAI. It also clarifies the contextual boundaries of existing empirical results, provides a differentiated operational path for the implementation of HCAI, as well as a clear direction and important reference for academic research and future practical applications of human-centered AI medicine.

## 1. Introduction

Artificial intelligence is reshaping the way healthcare services are delivered at an unprecedented speed, with its applications expanding across core healthcare domains including medical image analysis, disease risk prediction, personalized treatment recommendations and surgical assistance [1]. Synthesizing existing empirical studies [2,3,4], representative practical cases reveal the strengths of AI in diverse surgical scenarios: AI-assisted surgical guides for dental implantology deliver higher precision, stability and success rates compared with conventional tools and manual operations. AI-enabled platforms for neurosurgery support real-time prediction of intracranial aneurysm embolization, while advanced AI models applied to colorectal cancer surgery realize intraoperative real-time detection to boost clinical efficiency and safety. Collectively, these documented findings illustrate AI’s full-range empowering capacity covering preoperative diagnosis, intraoperative navigation and postoperative management [1,5].

However, a prominent “innovation–application gap” persists between AI technological advancement and large-scale clinical adoption [6]. As summarized in prior literature [7], this gap arises from organizational barriers, inadequate infrastructure, as well as widespread concerns over AI reliability and ethical risks. Existing research has pointed out that although AI is widely used in laboratories and startups, there are relatively few cases of it being truly integrated into healthcare provider organizations [6]. Based on a comprehensive review of relevant evidence [5,7,8], we categorize the key barriers into multiple dimensions: excessive costs, defective data quality and accessibility, limited algorithm adaptability, poor compatibility with electronic health record systems, mismatches with routine clinical workflows, and insufficient AI literacy and professional training among clinical staff. A regional institutional survey from Italy further validates such adoption disparities: 57% of the investigated healthcare facilities have not deployed any AI tools, and adopting institutions also adopt diversified implementation strategies [8]. In addition, ethical and legal challenges such as data privacy, security, algorithmic bias, and attribution of responsibility also seriously restrict the establishment of trust in AI and its widespread deployment [9,10].

Against this backdrop, the concept of “human-centered artificial intelligence” has emerged, emphasizing that the design, development, and deployment of AI systems must always be centered on human needs, values, and capabilities, and ensure that technology enhances rather than replaces human judgment and care [11,12]. HCAI is committed to designing AI systems that can enhance and improve human capabilities rather than replace humans. Its core is to combine “artificial intelligence” with “natural intelligence” to empower, amplify and enhance human performance [13]. This concept requires that the development and application process of AI fully incorporate the participation of multiple stakeholders, especially end users (such as clinicians and patients), and ensure the availability, credibility, and fairness of the technology through human-centered design principles [11,14]. For example, in AI-assisted surgery scenarios, studies have shown that human performance under AI assistance surpasses the performance of using AI alone or working alone, and this improvement exists in medical professionals of all professional fields and experience levels, challenging the common perception that AI mainly benefits junior clinicians [15]. This highlights the necessity of designing AI as a human partner rather than a replacement.

Design-driven innovation, as a systematic methodology, integrates the HCAI principle throughout the entire process from problem definition to solution implementation. Through iterative user participation and prototype testing, it creates usable, credible, and sustainable AI systems [11]. This approach emphasizes early and continuous user participation to understand actual clinical needs, workflows, and values, thereby designing solutions that are seamlessly integrated with clinical practice, easy to use and trustworthy [16]. For example, when developing AI clinical decision support systems, the design thinking approach, through continuous collaboration with non-technical experts (such as clinicians), can better address the challenges of model transparency and interpretability, thereby building more credible and usable tools [16]. This design-driven process helps bridge the gap between technological innovation and actual clinical adoption, ensuring that AI tools are not only technologically advanced but also ethically responsible and socially acceptable, ultimately achieving human-centered healthcare innovation.

Adopting a systematic narrative review methodology, this paper aims to address the inadequate theoretical systems, practical obstacles and incomplete ethical governance of HCAI in healthcare, establish a multi-dimensional analysis framework for medical HCAI, summarize the design-driven innovation path linking AI technology with clinical practice, and provide actionable solutions and references for academic research and practical application in this field.

## 2. Method and Framework

The study conducted a comprehensive literature search focusing on human-centered artificial intelligence, design-driven innovation, healthcare AI, implementation science and human–machine collaboration. Multiple mainstream academic databases were used, including PubMed, Web of Science, Scopus, and CNKI, covering peer-reviewed journal articles, conference papers and relevant academic reviews published from 2018 to 2025. Search keywords were combined in English and Chinese around the core themes of human-centered AI, explainable AI, generative AI, healthcare innovation and clinical implementation. Literature screening was performed in two stages: first, preliminary screening by title and abstract to exclude non-relevant papers, duplicate documents and grey literature; second, full-text reading to retain studies closely associated with the theoretical connotation, technical challenges, practical application and governance of HCAI. We excluded letters, commentaries and studies focusing purely on technical algorithm experiments without discussing human-centered design and clinical implementation. During literature analysis and synthesis, we categorized included studies by research theme, research field and research type. We prioritized peer-reviewed original studies and authoritative reviews for core argumentation, and differentiated the application scenarios and limitations of evidence from different domains. We integrated diverse theories and practical cases to form a logically structured analysis framework and innovation path, while objectively noting the differences and limitations of existing research findings across different healthcare fields.

**Figure 1:**
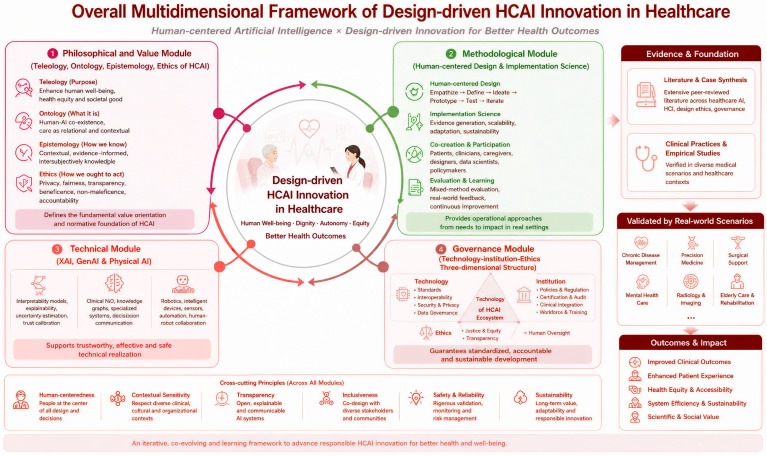
Overall Multidimensional Framework of Design-driven HCAI Innovation.

Specifically, the proposed multidimensional framework for design-driven HCAI innovation (Figure 1) consists of four interrelated core modules with clear boundaries: (1) Philosophical and value module (teleology, ontology, epistemology and ethics of HCAI), which defines the fundamental value orientation; (2) Methodological module (human-centered design and implementation science), which provides operational approaches; (3) Technical module (XAI, GenAI and physical AI), which supports technical realization; (4) Governance module (technology-institution-ethics three-dimensional structure), which guarantees standardized development. This framework is built on the collation and synthesis of a large body of peer-reviewed literature and practical cases across healthcare AI, and its rationality and adaptability are verified by existing clinical practices and empirical studies in different medical scenarios.

## 3. The Theoretical and Philosophical Foundations of Human-Centered Artificial Intelligence

### 3.1. Core Definition and Multidimensional Framework of HCAI

Human-centered Artificial Intelligence (HCAI) marks a fundamental shift in the development of AI in the healthcare field. Its core lies in transcending the technological paradigm of simply pursuing algorithmic efficiency and placing human well-being, values, and ethics at the center of system design [17]. This means that the development of AI tools must prioritize usability, understandability, fairness, and controllability to ensure that technology truly serves people rather than supersedes them [18]. In medical practice, HCAI requires AI systems to be committed to improving the quality of clinical decision-making, reducing the burden on medical staff, and ultimately improving patient experience and health outcomes, while maintaining the core values of doctor–patient relationships [19]. For example, AI-driven decision support systems should be designed to enhance rather than replace doctors’ professional judgment, thereby improving efficiency while maintaining the indispensable humanistic care in medical care [20].

Drawing on philosophical and theoretical literature on human-centered AI [21], we construct a comprehensive multi-perspective framework to support the development and governance of healthcare AI. This framework covers five core dimensions: teleology that defines human health as the ultimate goal rather than technological optimization; ontology that interprets human cognition, emotions and the instrumental nature of AI; epistemology that explores knowledge generation and verification via human–machine collaboration; axiology that integrates fairness, justice and transparency into algorithm design; and ethics that establishes practical ethical guidelines. Synthesizing the viewpoints from existing studies, this multidimensional philosophical framework sets the fundamental value benchmarks for HCAI. These philosophical guidelines do not remain at the theoretical level; instead, they directly constrain and guide the whole set of practical methods, technical design and governance rules involved in design-driven innovation, and run through the entire lifecycle of AI development and clinical application [22]. Therefore, HCAI is not only a technological concept, but also a comprehensive innovative path that integrates philosophical thinking, ethical considerations, and practical wisdom. To translate this human-centered philosophy into real-world healthcare practice, design-driven innovation acts as the core implementation vehicle, while human-centered design (HCD) and implementation science (IS) serve as two indispensable methodological pillars to advance HCAI deployment.

### 3.2. Driving Forces of the Paradigm Shift from Technology-Centric to Human-Centric Approach

The shift from a technology-centric to anthropocentric paradigm is primarily driven by the numerous real-world dilemmas arising from a purely technology-driven approach. In the past, the development model, which focused solely on algorithm performance, often resulted in AI tools being disconnected from actual clinical workflows, leading to low user acceptance and even safety and ethical risks [23]. For example, the opacity of “black box” algorithms undermines clinical trust, and AI systems lacking a deep understanding of medical contexts may not be able to effectively support complex decision-making processes [24]. Technology-centricity may also foster tools that prioritize management efficiency and are imposed on frontline medical staff from the top down, neglecting the actual needs of professionals and the core interests of patients, thereby exacerbating the tension between technology and humanism [23]. These challenges underscore the inherent limitations of technology-centric AI solutions that neglect human value considerations within complex healthcare ecosystems.

Secondly, sustained stakeholder engagement is critical to the successful implementation of healthcare AI [17]. Participation from clinicians, patients and management teams enables AI solutions to fit real clinical demands and user expectations [25]. Surveys of pregnant women and frontline nurses show that users prioritize doctor–patient empathy and ethical norms during AI adoption [25,26]. Stakeholder participatory design effectively narrows the divide between technical potential and real-world clinical application.

Finally, the growing ethical and regulatory pressures are another core driving force behind the paradigm shift. Widespread societal concerns about algorithmic bias, data privacy, security, and accountability compel AI innovation to be systematically integrated into human oversight and ethical review mechanisms [27]. Regulatory agencies emphasize the shift towards a human-centered healthcare model, requiring AI development to enhance rather than diminish human capabilities and be integrated into existing processes [28]. Building a sound AI governance framework has become a necessary mechanism for the safe transformation and application of AI systems in healthcare institutions [27]. At the same time, globally, especially in resource-constrained environments, the implementation of AI faces multiple obstacles such as infrastructure, data quality, a shortage of professional talent, and imperfect policies and regulations. These challenges further highlight the imperative to establish a systematic and humanistic implementation framework that fully incorporates technological, ethical, and societal considerations [18].

The above philosophical and driving factors jointly push the paradigm shift in healthcare AI, and the following chapters will further elaborate on how to translate these theoretical concepts into executable design and implementation methodologies.

## 4. The Methodologies and Application of Design-Driven Innovation

**Figure 2:**
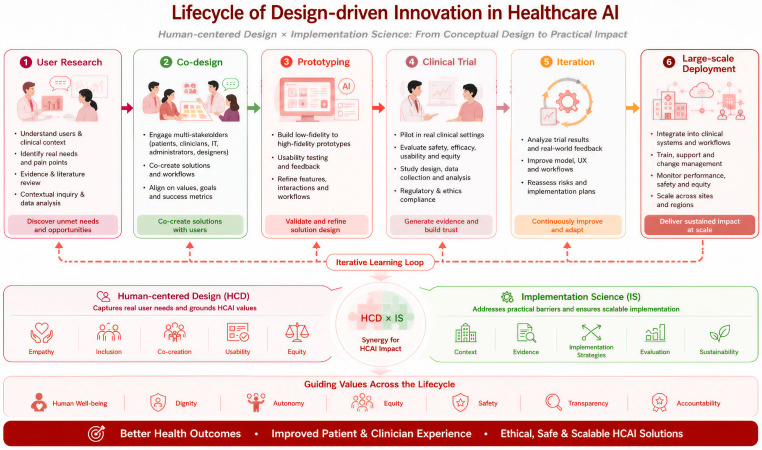
Lifecycle of Design-driven Innovation in Healthcare AI.

Design-driven innovation represents the core implementation paradigm of HCAI. On the one hand, human-centered design (HCD) grounds HCAI values by capturing real user demands throughout the innovation lifecycle; on the other hand, implementation science (IS) addresses practical barriers and guarantees the large-scale rollout of HCAI solutions in complex clinical environments. The combination of the two methodologies forms a complete logical chain from conceptual design to practical application for HCAI. This paradigm follows a standard workflow: user research, co-design, prototyping, clinical trials and full-scale rollout, with human needs as the core across the entire AI R&D and implementation lifecycle (Figure 2).

### 4.1. Human-Centered Design (HCD) Principles and Process

Human-centered design (HCD) is an iterative innovation approach that focuses on developing solutions by deeply understanding the needs, capabilities, and limitations of users (such as patients, doctors and nurses) in real-world environments [29]. This process typically involves multiple stages such as empathy research, problem definition, creative ideation, prototyping and user testing, emphasizing “design with users” rather than “design for users” [30]. In the healthcare field, the application of HCD has proven to significantly improve product usability and user acceptance.

A range of empirical cases have verified the practical value of HCD in healthcare scenarios [31,32,33]. A field study conducted in rural Tanzania adopted a five-stage HCD model to improve nurse-patient relationships in maternal and child healthcare, and summarized core operational strategies including user profiling, situational interviews, usability testing and participatory co-creation [30,31]. As evidenced by participant feedback, participatory co-creation stands out as an effective approach for joint solution design [30]. Further evidence from digital health research extends the application scope of HCD: relevant studies have applied this methodology to develop supportive tools for transplant nurses and optimize the service environment of neonatal intensive care units [32,33]. By sorting and analyzing these cross-scenario cases, we confirm that the iterative and participatory characteristics of HCD enable healthcare solutions to fully align with real user experiences and practical contexts.

### 4.2. The Role of Implementing Science (IS) in Bridging the Innovation Gap

Implementation Science (IS) provides systematic theories, models, and actionable strategies for promoting the integration and continued use of AI interventions in routine medical practice [34]. In practical deployment, it guides healthcare institutions to identify adoption barriers at individual, organizational and institutional levels, and formulate targeted solutions such as staff training, system adaptation and resource allocation to accelerate the stable application of HCAI solutions. Its core role is to identify and analyze multi-level barriers to innovation adoption and to design strategies to overcome these barriers, thereby bridging the “innovation gap” between research evidence and widespread practice. IS frameworks, such as the CFIR (Consolidated Framework for Implementation Research) and the RE-AIM (Reach, Effectiveness, Adoption, Implementation, Maintenance) framework, help researchers systematically examine the impact of individual (e.g., healthcare worker attitudes and skills), organizational (e.g., institutional culture and resource allocation), and systemic (e.g., policy environment and reimbursement mechanisms) factors on the adoption of AI tools [34]. For example, in the practice of integrating HIV with non-communicable disease services in Lusaka, Zambia, researchers applied the HCD approach to implementation science to adjust multi-level implementation strategies [35]. Through qualitative research with primary care and non-physician healthcare workers in four phases of exploration, conceptualization, analysis, and improvement, specific strategy sub-components were jointly designed, such as establishing NCD/HIV advocates and issuing weekly facility NCD drug bulletins, aiming to improve the adoption, appropriateness, and feasibility of interventions [35]. This reflects the complementarity of IS and HCD: HCD deeply understands user needs and jointly designs solutions, while IS provides a systematic framework to ensure that these solutions can be successfully embedded and sustained in complex healthcare systems [34]. By adopting hybrid research designs and adaptive implementation strategies, dynamic responses to AI can be achieved [36]. For example, some scholars have proposed the AHEAD (Approach to Human-centered, Evidence-driven Adaptive Design) framework. It bridges the gap between creative problem-solving and rigorous scientific validation. By marrying Human-Centered Design (HCD) principles with Health Services Research (HSR) methods, it empowers practitioners to build practical, sustainable healthcare interventions that truly resonate with [36]. In the development of AI monitoring systems, Vanderbilt University Medical Center adopted the HCD process and, through participatory design meetings and formative interviews, worked with diverse stakeholders to develop a prototype of the Vanderbilt Algorithmovigilance Monitoring and Operations System (VAMOS) to support the systematic and proactive monitoring of the lifecycle of medical AI tools [37]. This process identified the specific data views and functions required by different users, such as health system leaders, clinical experts, and technicians, ensuring that the monitoring system was closely integrated with organizational workflows and needs, which is key to successful implementation [37]. Therefore, implementation science, through its structured theories and methods, combined with human-centered design, provides a crucial path for the effective implementation and long-term maintenance of complex health technology innovations such as artificial intelligence in the real world.

## 5. Key Technologies and Their Human-Centered Design Challenges

As critical technical enablers for HCAI and design-driven innovation, explainable artificial intelligence (XAI), generative AI and physical AI tackle core technical obstacles in human-centered AI practice. Among them, XAI is a fundamental technical prerequisite for building clinical trust and realizing the practical value of HCAI.

### 5.1. Explainable Artificial Intelligence (XAI) and Clinical Trust Building

Explainable artificial intelligence (XAI) aims to make the decision-making process of AI models understandable and traceable to humans, which is crucial for building clinical trust in high-risk medical scenarios. Clinicians’ trust in AI systems is not generated automatically, but is influenced by a variety of factors, among which the transparency and explainability of the model are the core [38]. Multiple independent studies have confirmed a positive correlation between AI explainability and clinicians’ trust in AI systems [39]. After integrating and comparing relevant research findings [40,41], we find that the practical role of XAI is more complex than a single positive effect. Some clinical studies point out that while interpretability functions serve as a guarantee for clinical safety and trust, they may also bring potential drawbacks such as excessive cognitive burden. Meanwhile, evidence from ophthalmology research further indicates that the effect of XAI varies across clinical scenarios and clinician expertise levels [41].

Therefore, developing an intuitive visualization interface and ensuring that the interpretation itself is accurate and unbiased is the key to XAI design. In the surgical field, XAI is applied to preoperative risk assessment, surgical planning and postoperative monitoring, allowing surgeons to interpret and verify AI recommendations to make informed decisions [42]. However, generative AI currently lacks an inherent XAI mechanism, which is a major obstacle to its integration into clinical workflows [42]. To enhance trust, some studies have proposed that, in addition to interpretability, the transparency of model training information (including data sources and elements) and the clear consistency between model decisions and established clinical research results and guidelines are crucial to improving clinicians’ trust and acceptance of AI recommendations [43]. In addition, feature enhanced XAI models designed for specific clinical scenarios (such as Alzheimer’s disease diagnosis) that combine rule reasoning and example visualization can obtain 100% trust scores from doctors, significantly improving their confidence in using AI to assist in diagnosis [44]. These findings collectively indicate that well-designed XAI is the cornerstone for building clinical trust and realizing responsible innovation of AI in the medical field. To resolve the inherent trade-offs between model interpretability, computational performance and user cognitive burden, this study proposes a tiered XAI design framework tailored to clinical scenarios. For high-risk diagnosis and surgical scenarios, interpretability and accuracy are set as top priorities; for auxiliary administrative and follow-up tools, moderate explainability is adopted to balance operational efficiency. Meanwhile, designers need to differentiate explanation forms according to clinicians’ professional levels: simplified visual interpretation for frontline general practitioners, and in-depth logical deduction for senior specialists. This tiered framework provides actionable guidance to mitigate the dual risks of insufficient transparency and excessive cognitive load.

### 5.2. Generative Artificial Intelligence (GenAI) and Personalized Content Creation

Generative artificial intelligence (GenAI) can automatically generate text, images, and even 3D models based on context, which provides new possibilities for creating personalized patient education materials, rehabilitation training scenarios, or surgical simulation environments. In the field of diabetes care, GenAI can generate synthetic patient data, augmented datasets, simulate glucose-insulin dynamics, and develop virtual coaches and digital twins through deep generative models such as variational autoencoders, generative adversarial networks, transformers, and diffusion models, thereby addressing challenges such as data scarcity, patient variability, and personalization [45]. This capability makes it possible to provide patients with highly customized health management solutions. In extended reality (XR) environments, the “human-in-the-loop” approach, which combines GenAI with the creativity of human designers, can generate 3D assets that are more in line with the needs of immersive scenarios, such as those shown in the MineVRA framework. However, human-centered design requires a high degree of attention to the quality, security, cultural appropriateness, and potential biases of the generated content to prevent the generation of misleading or harmful information. This is especially important in complex areas such as rare diseases and genetic counseling. For example, a user study of facial phenotype analysis tools for genetic syndromes found that while deep learning and XAI-based diagnostic aids improved geneticists’ diagnostic accuracy and confidence, users remained skeptical of XAI support [46]. This serves as a warning that GenAI-generated content must undergo rigorous clinical validation and ensure that its interpretations are consistent with clinical reasoning. Furthermore, in scenarios such as mental health screening, the formation of trust is a sequential process influenced by model performance and its consistency with clinical reasoning [47]. Therefore, when applying GenAI to personalized content creation, strict quality control and safety protocols must be established, such as incorporating suicide risk markers into mental health AI models [47]. At the same time, it is necessary to ensure that the generated content is not only technically accurate but also culturally and socially appropriate, avoiding exacerbating health inequalities or producing discriminatory results. Ultimately, only by combining human professional judgment with the creativity of GenAI, supplemented by transparent interpretations and continuous evaluation, can truly patient-centered personalized medical content innovation be achieved. Given the multiple risks of content bias, misinformation and clinical inapplicability, we establish a three-layer risk governance framework for GenAI in medical scenarios: the primary layer is pre-generation control via diversified training datasets to reduce inherent bias; the secondary layer adopts real-time clinical validation by medical professionals to filter misleading content; the tertiary layer conducts post-use feedback and iterative optimization. When balancing content personalization and universal safety, medical GenAI should follow the principle of “safety first, personalized optimization second”, and adjust content generation intensity according to disease types and patient groups, especially for rare diseases and mental health fields with high risk sensitivity.

### 5.3. Human–Machine Collaboration in Physical Artificial Intelligence and Robotics

Physical AI enables robots to perceive, reason, and act in dynamic environments, and can be used in the nursing field for logistics, cleaning, and even assisting in direct nursing tasks, such as moving patients. The key to human–machine collaborative design is to clearly define roles and let robots act as “silent partners” or assistants, aiming to enhance rather than replace the professional judgment and humanistic care of nursing staff. Studies have shown that nurses’ trust in AI plays a mediating role in the relationship between AI uncertainty and AI capabilities [48]. This means that when designing physical AI systems, the psychological safety needs of end users (such as nurses) must be considered, and uncertainty should be reduced through transparent decision-making processes, providing AI clinical cases and training, thereby building trust [48]. This trust is crucial for robots to be successfully integrated into nursing teams and achieve effective collaboration. Challenges include ensuring the intuitiveness of the interface, dealing with uncertainty in unstructured environments, addressing ethical issues (such as safety, privacy, and emotional dependence), and avoiding exacerbating health inequalities due to differences in technology accessibility [38,49]. To optimize human–machine collaboration, this study puts forward a role-partitioning design framework: physical AI robots undertake repetitive, high-intensity and low-risk auxiliary work, while medical staff retain core clinical judgment, emotional communication and emergency disposal authority. This role division clarifies responsibilities and avoids functional overlap or excessive technological substitution. In terms of design trade-offs, system robustness in unstructured clinical environments takes precedence over full automation; meanwhile, accessibility configuration should be adapted to different medical institutions to narrow the health equity gap. Through iterative user testing and targeted training, caregivers can gradually build trust, ultimately achieving complementary advantages between humans and machines to improve the overall quality and efficiency of care.

**Figure 3:**
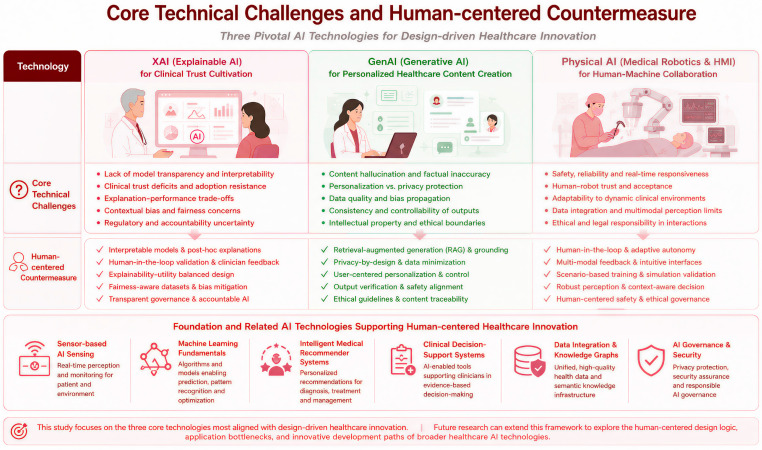
Core Technical Challenges and Human-centered Countermeasures.

To sum up, this section systematically analyzes the human-centered design challenges of three pivotal AI technologies for healthcare innovation, covering explainable artificial intelligence for clinical trust cultivation, generative artificial intelligence for personalized healthcare content creation, and human–machine collaboration mechanisms for medical physical intelligence and robotics. Beyond the aforementioned core technological categories, a diverse array of emerging and foundational AI technologies also exert crucial influences on modern healthcare practices. These key technological domains include sensor-based AI sensing techniques, fundamental machine learning algorithms, intelligent medical recommender systems, and various AI-enabled clinical decision-support technologies, all of which serve as essential technical pillars for advancing human-centered healthcare AI innovation. Given the manuscript’s thematic focus and structural arrangement, this study concentrates on the above three representative technological directions that are most closely aligned with design-driven healthcare innovation, without expanding on the detailed design characteristics and practical challenges of other auxiliary AI technologies. Subsequent research can build on this study’s framework to further explore the human-centered design logic, application bottlenecks, and innovative development paths of these diversified healthcare AI technologies, thereby enriching and improving the theoretical and practical system of design-driven human-centered artificial intelligence in healthcare (Figure 3).

## 6. Adoption Willingness and Barrier Analysis for Diverse Users

### 6.1. The Attitude, Preparedness, and Workflow Integration of Medical Staff

Synthesizing global survey evidence [50,51,52], most healthcare professionals hold a positive attitude towards AI adoption in clinical practice. Nevertheless, existing cross-regional and cross-professional studies reveal obvious heterogeneity in such attitudes [50,53,54]. These divergences are closely tied to local medical systems, digital infrastructure and talent training systems: surveys from Turkey and Egypt, which are in middle-income regions, show that age and gender are prominent influencing factors, largely due to uneven digital literacy across groups, while research conducted in the UK, a high-income region with mature digital healthcare systems, places more emphasis on the matching degree between AI and professional work scenarios rather than demographic characteristics. Such regional differences mean that relevant research conclusions cannot be directly generalized globally. Targeted promotion strategies should be formulated according to local conditions: regions with weak digital foundations need to prioritize staff training, while mature medical systems focus on workflow adaptation. Regional surveys conducted in Turkey and Egypt reflect differences between occupational groups, genders and age cohorts, while research on hearing health practitioners in the UK further proves that professional backgrounds also shape perceptions of AI. We summarize these divergent findings and analyze the underlying influencing factors in the following discussion. These findings collectively suggest that acceptance of AI is influenced by a complex interplay of factors such as age, educational background, history of technology exposure, gender, and professional role.

Despite positive expectations, the adoption of AI in clinical practice still faces multiple obstacles. Primary concerns include concerns about workflow disruption and doubts about the reliability of algorithms [51]. Healthcare professionals are generally worried that AI systems may not be able to integrate seamlessly into existing workflows and may instead increase operational complexity [55]. Deeper obstacles involve fears of weakened doctor–patient relationships and perceptions of threatened clinical autonomy [56]. Many healthcare professionals worry that the intervention of AI will reduce interpersonal interaction with patients, weaken empathy-based care, and question who should bear the ultimate responsibility in AI-assisted or AI-led decisions [56,57]. In addition, concerns about data privacy, algorithmic bias, and potential burnout from mastering AI-related skills are also prevalent [56]. These obstacles together constitute the psychological and operational challenges that need to be overcome in the process of AI integration.

Successful integration strategies emphasize a gradual approach, starting with automating administrative tasks and gradually transitioning to clinical decision support. Studies have shown that the initial phase should focus on areas that can directly reduce the administrative burden on healthcare workers, such as automated documentation and appointment management [58]. For example, in dermatology, AI-driven automated medical record tools have been shown to optimize workflows and reduce the risk of physician burnout [58]. This approach, from simple to complex, helps build trust and demonstrate the practical value of AI. A key step is to ensure that the AI system is seamlessly integrated with existing electronic health record systems to minimize workflow disruptions and improve user experience [59]. For example, integrating convolutional neural networks used to detect intracranial hemorrhage into clinical workflows has enabled near real-time processing and high-precision diagnosis, demonstrating the feasibility of technology integration [59]. Ultimately, successful integration requires accompanying training and education to improve healthcare workers’ knowledge of AI and their operational confidence, thereby overcoming adoption barriers and realizing the potential of AI tools in improving patient safety and work efficiency [60,61].

### 6.2. Accessibility and Inclusivity Design for Patients and People with Disabilities

For patients and people with disabilities, one of the strongest predictors of adoption of health-related technologies is perceived usefulness, i.e., how much a technology is perceived to improve their health or quality of life [62]. However, even with perceived usefulness, significant barriers to adoption persist. These barriers include accessibility challenges due to physical and cognitive limitations, such as the difficulty users with visual or motor impairments may have to operate standard interfaces [63]. Secondly, high technology costs, insufficient training support, and the pervasive digital divide exclude people with limited economic means or low digital literacy [63]. In resource-constrained environments, such as Pakistan, despite doctors’ high interest in using AI to improve professional efficiency, challenges such as lack of funding, insufficient training, and provider resistance severely limit the integration of AI [64]. These structural barriers make technology accessibility far from equitable. The severity of such barriers varies significantly across groups and regions: patients with physical or cognitive impairments face the most prominent accessibility challenges; in low- and middle-income areas, economic thresholds and insufficient training become the primary obstacles, whereas in developed regions, patients are more concerned about privacy and emotional alienation. Hence, inclusive design solutions cannot adopt a one-size-fits-all model, and their generalization is restricted by population characteristics and regional development levels.

In addition to technical and economic barriers, socio-psychological factors also play a key role in shaping patients’ negative adoption of medical AI. Fear of safety risks is a core concern, with patients worried that AI systems may make incorrect diagnoses or treatment recommendations, thereby endangering their health [7]. Social stigma is another important factor, especially for people with disabilities who use assistive technologies, who may worry about being labeled [56]. In addition, concerns about reduced interpersonal interaction are particularly prominent; many patients value the trust- and empathetic relationship with doctors and worry that the intervention of AI will make medical care dehumanizing and indifferent [56,65]. A systematic review pointed out that patients and the public are skeptical about whether AI can provide empathetic care and stressed that the development and application of AI requires patient participation and clinical validation [56]. These socio-psychological factors profoundly affect users’ trust in the technology and their eventual willingness to adopt it.

Inclusive design is the fundamental way to address the above challenges, and its core principles are to prioritize accessibility, affordability, and trust building. This means that the involvement of users with disabilities and diverse patient groups must be actively incorporated from the design stage to ensure that the product meets their real needs [66]. For example, developing a user interface that supports multiple interaction modes (such as voice control and eye tracking) can greatly improve accessibility for users with physical limitations [63]. At the same time, it is crucial to build trust through transparent design processes, clear communication, and strong data privacy protection measures [67]. A study on the application of AI in breast cancer screening found that while female participants welcomed AI-driven thermal imaging technology as a non-invasive alternative, they also expressed concerns regarding technological novelty, insufficient clinical validation, inherent algorithmic bias, and data privacy issues, and stressed the need for rigorous supervision and transparent interpretation from medical professionals [68]. Therefore, inclusive design is not only a technological adaptation, but also a systematic project involving ethics, communication, and continuous participation, aiming to ensure that technological innovation can benefit all people, including the most marginalized patients and people with disabilities.

## 7. Strategic Priority Assessment and Governance Framework

Complementary to the methodologies and technical support mentioned above, a sound three-dimensional governance structure defines the bottom line and long-term development rules for HCAI. Together with human-centered design, implementation science and key AI technologies, it constitutes the complete multidimensional framework of design-driven HCAI innovation.

### 7.1. Application of Multi-Criterion Decision-Making Model in Innovation Prioritization

In an environment where medical AI innovation resources are limited, it is crucial to systematically evaluate and select projects. Multi-criteria decision analysis (MCDA) models provide a structured framework for this purpose, enabling decision-makers to weigh conflicting goals and criteria [69]. For example, the Analytic Hierarchy Process (AHP), as a mature MCDA method, has been used to perform multi-criteria analysis on innovative medical devices. By comparing the performance of different options under specific criteria and sub-criteria, the most advantageous solution is identified to enhance the robustness of the decision-making process [70]. In addition, models such as the Technique for Order of Preference by Similarity to Ideal Solution (TOPSIS) have also been applied to technology adoption scenarios. For example, in Active Assisted Living (AAL) systems, TOPSIS combined with fuzzy methods has been used to select the optimal solution from a series of classification algorithms to support research on the implementation of care for the elderly [71]. The application of these models enables medical institutions to go beyond single technical indicators and make more comprehensive considerations. The evaluation dimensions need to cover a wide range of key aspects of medical innovation. Studies have shown that human-centered artificial intelligence and automation are often regarded as the most effective enabling factors for improving the quality of healthcare [72].

To illustrate its practical operation in real healthcare decisions, we take the institutional selection of clinical AI diagnostic tools as a typical application scenario. When a hospital plans to introduce multiple AI-assisted diagnosis products, decision-makers can apply AHP to layer evaluation targets, primary criteria and detailed sub-indicators step by step. At the top level is the overall goal of selecting a practical and safe AI solution; the primary criteria cover clinical effectiveness, safety, implementation cost, system compatibility and long-term maintenance capability. Each primary criterion is further decomposed into quantifiable sub-indicators, such as diagnostic accuracy, adverse event rate, procurement cost, and compatibility with local electronic health record systems. Decision-makers assign differentiated weights to each indicator according to institutional priorities—for tertiary hospitals focusing on high-risk diagnosis, higher weights are given to safety and clinical effectiveness; for primary care facilities with tight budgets, cost and operability become dominant factors. After scoring all candidate AI products against unified indicators, the model outputs a clear ranking to support final project selection, which solves the dilemma of subjective and one-sided decision-making in traditional product screening.

A standardized MCDA framework usually includes multiple evaluation dimensions, such as clinical effectiveness, clinical safety, innovativeness, disease severity, implementation capability, and cost [69]. In the evaluation studies of implantable medical devices in China, clinical safety and cost were given the highest weight by stakeholders, followed by implementation capability and clinical effectiveness, while innovativeness and disease severity were given lower weights [69]. This reflects that in real-world decision-making, safety, economic feasibility, and implementation capability are often the core considerations that take precedence over pure technological novelty. Other important dimensions include patient-centered digital healthcare, sustainable healthcare practices, workforce adaptation, and ethical and regulatory compliance, which together constitute a comprehensive evaluation system.

The core value of this model lies in helping institutions make structured decisions in complex environments, balancing technology readiness, ethical requirements, and patient-centered values. By assigning weights to different criteria and conducting systematic scoring, the MCDA model can transform subjective judgments into comparable and reproducible decision-making criteria [70]. Taking AI research and project approval for epilepsy management as another practical case [73], medical institutions need to balance diagnostic performance, patient benefit, health equity and resource input. The MCDA model quantifies these multiple conflicting objectives: projects that can improve diagnosis accuracy and reduce disparities among patient groups will obtain higher comprehensive scores, even if they require moderate resource investment. This operational logic allows managers to avoid prioritizing technical novelty alone and allocate limited resources to AI initiatives that deliver maximum clinical and social value. Effective prioritization models can guide resources toward projects that meet urgent clinical needs, align with institutional strategic goals (such as sustainability and workforce empowerment), and have manageable risks [72] thereby supporting healthcare systems in making informed choices in a rapidly changing innovation environment.

### 7.2. Three-Dimensional (Technology-Institutional-Ethics) Governance Structure

Effective AI governance is a complex, multi-dimensional undertaking that requires the coverage of three interconnected and mutually supportive dimensions: technology, institutions, and ethics. This three-dimensional framework is the foundation for ensuring responsible innovation and sustainable development of AI in the healthcare field. The focus of governance in the technology dimension is to ensure the reliability, transparency, and fairness of AI systems. This requires improving the transparency of algorithms through technologies such as explainable AI (XAI) so that the clinical decision-making process is no longer a “black box” [74]. At the same time, culturally aware design is also crucial, requiring algorithm development to take into account the socio-cultural backgrounds of different groups in order to promote algorithmic fairness and reduce bias [75]. For example, AI-driven facial analysis systems trained solely on Western-centric aesthetic standards may exacerbate bias in plastic surgery [76]. In routine technical governance of medical AI, clinical regulatory departments apply this dimension to conduct regular algorithm audits. For instance, before the official launch of an AI imaging diagnosis system, auditors use XAI tools to trace its decision logic, verify data diversity across different age, gender and ethnic groups, and detect potential demographic bias. Unqualified algorithms will be required for iterative optimization before clinical deployment, which realizes real-time technical risk control in practice.

The institutional dimension provides necessary rules and infrastructure guarantees for the governance of artificial intelligence. This first requires the establishment of a privacy-protected data platform to strictly protect patient data sovereignty and privacy while promoting data flow to support innovation [77]. Secondly, it requires the construction of a risk-based regulatory framework, such as the priority review and special licensing system established by some countries for innovative medical devices [78]. In addition, transnational governance mechanisms coordinate data and regulatory standards [74]. Practical application: For regional healthcare authorities, this institutional dimension guides the formulation of unified management rules for medical AI. Taking a provincial medical AI promotion project as an example, the authority first builds a unified medical data platform with strict access control to protect patient privacy; then classifies AI products by risk level and adopts differentiated approval procedures: high-risk diagnostic AI undergoes strict full-cycle review, while low-risk auxiliary AI tools apply simplified filing mechanisms. This tiered institutional arrangement balances innovation incentives and risk prevention in real governance work.

**Figure 4:**
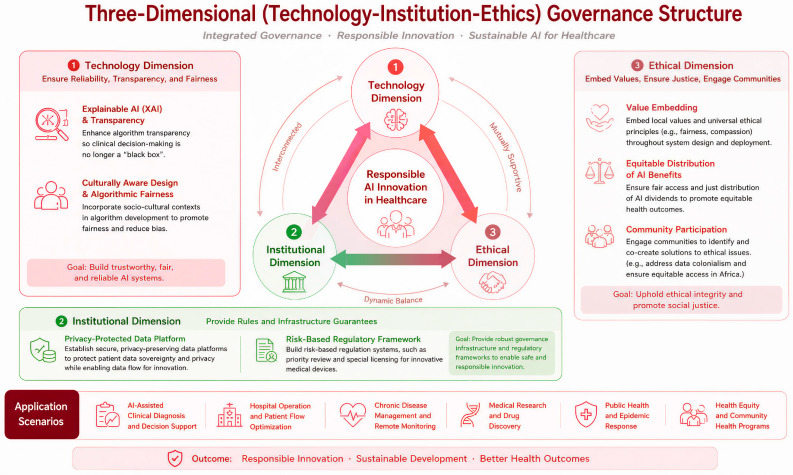
Three-Dimensional (Technology-Institution-Ethics) Governance Structure.

The ethical dimension is the soul of AI governance, requiring the deep embedding of local values and universal ethical principles into the entire process of system design and deployment. This includes ensuring that values such as fairness and compassion are reflected in the algorithm and paying attention to the fair distribution of AI dividends in order to maintain just health outcomes [79]. Community participation is considered an important way to identify and collaboratively solve related ethical issues. For example, when conducting AI health research in Africa, community participation is necessary to address challenges such as data colonization and equitable access [80]. When rolling out public health AI services such as intelligent chronic disease management, the ethical dimension runs through the whole process. Governance teams invite patients, community representatives and clinical staff to participate in joint evaluation, inspect whether the service design ignores vulnerable groups such as the elderly and low-income populations, and assess whether AI application widens health disparities. For problematic designs, adjustments are made to ensure equitable access to AI healthcare services for all groups. Ethical governance also requires attention to the impact of AI on environmental sustainability and to promote the development of smart medical solutions that consume less resources and are more environmentally friendly [79]. Ultimately, a sound ethical governance structure can guide the development of AI not only to pursue efficiency and precision, but also to dedicate itself to improving the health and well-being of all mankind and social equity (Figure 4).

## 8. Interdisciplinary Collaboration and Co-Creation of Ecosystems

### 8.1. Stakeholder-Driven Co-Creation Model

Successful human-centered AI (HCAI) innovations rely heavily on deep collaboration across disciplines and multiple roles. Research shows that early and sustained involvement of key stakeholders such as clinicians, AI engineers, designers, ethicists, patient representatives, and managers is crucial to ensuring that AI tools are practical, sustainable, and ultimately successfully integrated into clinical workflows [81]. A qualitative study of the UK National Health Service (NHS) found that while healthcare workers were open to AI, they were also concerned that it might increase their workload or lead to misdiagnosis, highlighting the importance of taking into account the real needs of frontline clinicians early in the development process [82]. Collaborative approaches such as co-creation workshops, joint design meetings, and prototyping can help break down disciplinary barriers, jointly define clinical problems, explore solutions, and assess their potential impact [83]. For example, in the reform attempt to integrate AI algorithms into the Scottish National Breast Cancer Screening Service, a stakeholder analysis, through focus groups and interviews, brought together the views of 32 participants from 14 subgroups, including clinical, management, and technical personnel, to discuss the advantages and disadvantages of AI in improving service efficiency and potentially exacerbating diagnostic inequalities, and to develop strategies for managing key stakeholders [84]. This multi-stakeholder engagement model is crucial for identifying and addressing a range of complex issues, from technical performance to ethics and regulation [85]. Of particular note is the significant potential of the nursing community as an important stakeholder in driving innovation in nursing practice and integrating digital health and AI. A discussion paper suggests that the nursing community should participate as an active stakeholder in the healthcare AI governance ecosystem, utilizing its teaching, research, and service functions to ensure that AI solutions are closely integrated with the real needs of nursing work, thereby safeguarding health-related human rights [86]. Community engagement is also an indispensable but often overlooked part of the co-creation model. A scoping review found that of the approximately 10,880 articles describing AI applications in healthcare, only 21 (0.2%) involved community engagement, and most of them were limited to the use of community data. Only one article described the inclusion of community stakeholders in the application design [87]. This suggests that extending the co-creation of healthcare AI innovations from hospitals to a broader community setting is of great value for optimizing the universality and acceptability of solutions.

### 8.2. Cultivating a Human-Centered AI Innovation Culture and Leadership

Promoting human-centered AI innovation requires fostering a culture within organizations that encourages experimentation, tolerates failure, and empowers frontline healthcare workers to generate innovative ideas and participate in the design process [81]. This culture is fundamental to overcoming many obstacles in AI implementation. A qualitative study of healthcare AI implementers in Australia identified resource constraints, lengthy implementation timelines, and insufficient stakeholder involvement as major obstacles, and a supportive organizational environment is crucial to addressing these challenges [88]. In this context, leadership plays a particularly important role. Leaders need to play a key role in fostering accountability, improving organizational readiness and ethical management [89]. They need to advocate a vision that aligns AI with human values, invest in retraining staff, and establish governance structures that support responsible innovation [90]. For example, when procuring innovative clinical AI products, leaders need to understand and address challenges such as uncertainty about the value of AI products, inefficiencies in processes, and sustainability, and promote responsible innovation by designing reasonable procurement frameworks [91]. At the same time, leadership also needs to pay attention to and mitigate the risk of burnout that AI may bring, prioritizing AI technologies that are aligned with stakeholder values and committed to “rehumanizing” medical practices to restore healthcare workers’ sense of mission and efficacy [92]. To fundamentally prepare the future healthcare workforce, reform of the education system is imperative. Medical education worldwide needs to incorporate design thinking, AI literacy, ethics and interdisciplinary collaboration skills into the core curriculum of medicine, nursing and public health [86]. A hybrid approach study of 128 medical students in 48 countries found that while students showed knowledge and interest in the clinical application of AI, very few had received formal instruction in the field [93]. Students generally supported the inclusion of AI instruction in the core curriculum and wanted to learn about clinical applications, algorithm development, coding and algorithm evaluation, while also suggesting innovative teaching methods such as hackathons and multidisciplinary education (e.g., collaboration with computer science students) [93]. This educational transformation is not only aimed at students but also at working healthcare professionals. Studies have shown that while healthcare professionals have a positive overall understanding of AI, they generally lack sufficient knowledge and have limited opportunities to build relevant skills, which restricts the acceptance and integration of AI [94]. Therefore, developing practical AI courses and providing continuous skills retraining for all medical practitioners, including doctors and nurses, is the only way to cultivate medical leaders and practitioners who can handle the complexities of the AI era [95]. Ultimately, only through a multi-pronged approach of cultural shaping, leadership promotion, and educational reform can we build a responsible and sustainable healthcare ecosystem that supports human-centered AI innovation.

## 9. Future Research Directions and Challenges

### 9.1. Deepening the Measurement and Evaluation Framework

In the future, the evaluation framework for human-centered artificial intelligence (HCAI) needs to move beyond traditional clinical accuracy or efficiency indicators and shift towards a more comprehensive measurement of value. This requires the development of new evaluation tools that take into account dimensions such as health equity, social impact, user experience and long-term sustainability [96]. For example, the World Health Organization’s ethical and governance guidelines emphasize that the evaluation of AI technologies such as large multimodal models must examine their impact on social equity and ethical compliance [96]. To systematically evaluate the comprehensive value of HCAI innovation, it is essential to establish standardized “human-centered” indicators. These indicators should cover multiple aspects such as user trust, workflow integration, decision support quality and ethical compliance [97].

Based on a systematic narrative review of XAI research in healthcare [97], we extract core evaluation indicators including user satisfaction, trust level, task performance and correctability, which lay a foundation for establishing human-centered assessment criteria. Meanwhile, by analyzing the lifecycle evaluation logic of the HEAAL framework and other mainstream tools [98], we discuss the feasibility of applying multi-dimensional equity assessment to medical AI evaluation systems. The Lifecycle assessment aims to evaluate the potential impact of AI solutions on health equity throughout the entire lifecycle, covering multiple assessment areas such as accountability, fairness, purpose applicability, reliability and effectiveness, and transparency [98].

This suggests that future assessments need to be a process-oriented framework with step-by-step procedures to guide healthcare institutions on how to mitigate the potential risks of AI solutions exacerbating health inequalities [98]. Similarly, quantitative assessments of healthcare AI policy texts show that the focus of policies and the integrity of the assessment system are advantages, but policy continuity and the operability of tools still need to be strengthened [99]. Therefore, deepening the measurement and assessment framework means shifting from a single performance evaluation to a multi-dimensional, full-lifecycle, and dynamically adaptable comprehensive assessment system to ensure that AI innovation truly serves the comprehensive health and well-being of people.

### 9.2. Addressing the Core Challenges at the Data and Algorithm Levels

At the data and algorithm level, human-centered medical AI faces a series of core challenges. The primary issue is how to mitigate historical bias in training data to prevent algorithmic discrimination [100]. Studies have shown that biases in AI systems can be categorized as historical/representative bias, selection/measurement bias, algorithmic/optimization bias, and feedback/emergent bias, which may replicate and amplify structural inequalities [100]. For example, a Turing test-inspired approach has been used to analyze the pervasive biases in AI-based medical imaging, which helps detect whether algorithms are biased towards specific types of cases [101]. Secondly, overcoming model overfitting to ensure generalization ability across different populations and institutions is crucial. Many AI systems still face an “AI gap” in terms of validation, regulation, and safe implementation, which needs to be bridged to realize their full potential [102]. Improving the transparency of medical AI decision-making to address the “black box” problem is equally urgent. Research on explainable artificial intelligence (XAI) aims to address the lack of a comprehensive and accepted explanatory framework and standardized methods for evaluating the effectiveness of explanations [97]. To address these challenges, it is necessary to promote the construction of a high-quality, diverse, and FAIR-compliant medical data sharing ecosystem. For example, by combining crowdsourcing frameworks with data quality assurance measures, the challenge of scarcity of high-quality labeled data can be effectively alleviated in resource-constrained environments and used to train large language models in the medical field [103]. At the same time, knowledge graphs, federated learning and other technologies can be used to enhance the value of data while protecting privacy. In low-resource environments, large multilingual LLMs can be transformed into lightweight, task-optimized small language models (SLMs) through knowledge distillation frameworks, such as the application of AraSum in Arabic medical summarization, demonstrating a feasible path to improve performance while protecting data privacy and reducing computational costs [104]. These technologies and methods together constitute the core strategies for addressing data bias, improving model generalization ability and transparency, and are the cornerstone for building fair, reliable, and trustworthy medical AI systems.

### 9.3. Evolution Towards Safe and Sustainable Design (SSbD)

By the EU’s “Safe and Sustainable Design” framework, the innovation path of medical AI should incorporate safety, sustainability, and ethical impact into the design considerations from the very beginning, so as to achieve the evolution towards safe and sustainable design. This requires the use of new methods to assess the environmental and social impacts of AI systems throughout their entire lifecycle, ensuring that technological innovation is responsible and future-oriented and aligned with broader sustainable development goals [105]. For example, empirical analysis of the application of artificial intelligence of things (AIoT) technology in the medical field shows that AIoT technology represents a paradigm shift toward sustainable healthcare systems, creating a self-reinforcing ecosystem across economic, organizational, and social dimensions, where improvements in any one dimension enhance outcomes across all areas, thereby comprehensively promoting the achievement of the sustainable development goal of “good health and well-being” [105]. This lifecycle perspective is also reflected in the evaluation of AI solutions, such as the HEAAL framework. This framework examines five equity assessment domains (accountability, fairness, fitness for purpose, reliability and validity, and transparency) across eight key decision points throughout the AI adoption lifecycle: (1) Identify and prioritize a problem, (2) Define AI product scope and intended use, (3) Develop success measures, (4) Design AI solution workflow, (5) Generate evidence of safety, efficacy, and equity, (6) Execute AI solution rollout, (7) Monitor the AI solution, and (8) Update or decommission the AI solution. It highlights full accountability for AI systems from initial design to final deployment and retirement [98]. In terms of environmental sustainability, the evaluation of climate-aware indicators for radio informatics and artificial intelligence and the exploration of potential radioecological labels reflect a concern for the environmental footprint of AI technology [106]. At the same time, ensuring the safety of AI systems involves continuous monitoring of algorithm transparency, reliability, and ethical compliance. A detailed case study of AI applications for clinical decision support shows that critical evaluation using a medical ethics approach can identify issues related to patient care, such as potentially deceptive promises, lack of patient and public engagement, etc. [107]. Therefore, the evolution toward SSbD means that the development and deployment of medical AI must adopt an integrated framework that combines statistical diagnostics with governance mechanisms to achieve bias mitigation and impact management throughout the AI lifecycle [100]. This requires policymakers, developers and healthcare institutions to work together to establish an agile, transparent and ethically oriented oversight system that incentivizes innovation while ensuring patient safety and health equity, so that AI technology can truly enhance rather than harm healthcare outcomes.

## 10. Conclusions

Design-driven innovation towards “human-centered artificial intelligence” relies on a complete multidimensional framework composed of value orientation, methodologies, core technologies and governance rules. This integrated system points to a fundamental direction for the development of AI in the healthcare field. Its core value lies in the fact that it is not a simple patching of existing technological paths, but rather a driving force for a profound paradigm shift—from pursuing isolated algorithmic performance to building an integrated system based on human well-being, clinical workflows, and universal ethical values. The success of this shift depends on the synergy of multi-dimensional methodologies: human-centered design ensures that technology aligns with real-world needs; scientific implementation guarantees that innovation can effectively integrate into complex healthcare environments; explainable AI (XAI) technology is the cornerstone of building clinical trust; and a strategic governance framework defines the boundaries and trajectory for the healthy development of the entire ecosystem. These elements must be united and driven by deep co-creation among interdisciplinary teams and strong institutional leadership.

Currently, the road to this ideal vision remains fraught with challenges. Technically, the “black box” nature of algorithms and potential biases threaten their impartiality and reliability. Data-wise, inconsistent quality, insufficient representativeness, and accessibility barriers constrain the inclusive value of AI. Organizationally and culturally, ingrained work patterns, skills gaps, and resistance to change are invisible obstacles that must be overcome for the technology to be implemented. Addressing these challenges requires systemic investment that goes beyond the technology itself: continuous investment in inclusive design processes to allow diverse voices to participate in shaping AI; establishing and iterating dynamic ethical and governance frameworks to regulate development and mitigate risks; vigorously promoting capacity building among healthcare professionals, patients, and administrators to improve digital health literacy; and strengthening evaluation research on actual impacts and long-term effects to drive optimization through evidence.

Looking to the future, the ultimate success of medical AI will no longer be marked by breakthroughs in a single technological metric, but rather by its practical effectiveness in enhancing human professional capabilities, maintaining the irreplaceable human connection in healthcare, and promoting health equity. This means we must carefully balance technological innovation, forward-looking policies, and profound humanistic concern. Technological innovation is the engine, policy is the steering wheel, and humanistic concern is the destination and the measure of value. Only by maintaining a dynamic balance and organic uniformity among technological progress, policy guidance, and humanistic orientation can we ensure that AI truly serves humanity, empowers medicine, and ultimately leads us towards a more equitable, efficient, and compassionate future of smart healthcare.

## 11. Limitations and Future Research

This systematic narrative review has several inherent limitations that need to be acknowledged. First, in terms of literature selection, this study retrieved publications only from four mainstream academic databases (PubMed, Web of Science, Scopus and CNKI) with a fixed publication timeframe from 2018 to 2025. We excluded grey literature, conference abstracts, non-English monographs and pure technical experimental papers without discussion on human-centered design and clinical implementation. This retrieval and screening strategy may introduce selection bias and result in the omission of valuable regional studies, fragmented practical experiences and emerging technical viewpoints.

Second, regarding the research scope, this review primarily focuses on human-centered AI and design-driven innovation in mainstream clinical healthcare scenarios. It does not conduct in-depth discussion and analysis on occupational health, physical health intervention, mental health services, public health governance and disability care, which limits the generalizability of the proposed theoretical framework and innovation path.

Third, as a systematic narrative review, this work mainly relies on qualitative literature collation, thematic integration and framework construction. We did not conduct quantitative meta-analysis or original empirical verification for the proposed multidimensional framework, so the quantitative validity of relevant conclusions remains to be further tested.

For future research, we will expand literature sources and retrieval scope to cover more databases and multilingual studies, enrich research scenarios across diversified healthcare fields, and combine empirical investigation and quantitative analysis to further optimize and validate the design-driven innovation path of human-centered artificial intelligence in healthcare.

## Data Availability

No new data were created or analyzed in this study. Data sharing is not applicable to this article.

## References

[1] Guo C., He Y., Shi Z., Wang L. (2025). Artificial Intelligence in Surgical Medicine: A Brief Review. Ann. Med. Surg..

[2] Gupta S., Yadav D., Anubhuti A., Siddiqui L.T., Rudrappa D., KalamAzad A., Sahoo A. (2025). AI Assisted Surgical Guides for Dental Implant Placement. Bioinformation.

[3] Williams K.A., Podgorsak A.R., Bhurwani M.M.S., Rava R.A., Sommer K.N., Ionita C.N. (2021). The Aneurysm Occlusion Assistant, an AI Platform for Real Time Surgical Guidance of Intracranial Aneurysms. Proc. SPIE—Int. Soc. Opt. Eng..

[4] Li L., Xuan B., Song X., Tian Y., Meng X., Wen J., Zheng T., Liu C. (2025). AI-Assisted surgical vision: Evaluating YOLOv8 and YOLOv12 for real-time detection in colon cancer surgery. Front. Surg..

[5] Li M.X., Deng R.S., Yuan C.H. (2025). The Application of Artificial Intelligence in Enhancing the Safety of Pancreatic Surgeries. Zhonghua Wai KE ZA Zhi [Chin. J. Surg.].

[6] Davenport T.H., Glaser J.P. (2022). Factors Governing the Adoption of Artificial Intelligence in Healthcare Providers. Discov. Health Syst..

[7] Ahmed M.I., Spooner B., Isherwood J., Lane M., Orrock E., Dennison A. (2023). A Systematic Review of the Barriers to the Implementation of Artificial Intelligence in Healthcare. Cureus.

[8] Ardito V., Cappellaro G., Compagni A., Petracca F., Preti L.M. (2025). Adoption of Artificial Intelligence Applications in Clinical Practice: Insights from a Survey of Healthcare Organizations in Lombardy, Italy. Digit. Health.

[9] Khan M.M., Shah N., Shaikh N., Thabet A., Alrabayah T., Belkhair S. (2025). Towards Secure and Trusted AI in Healthcare: A Systematic Review of Emerging Innovations and Ethical Challenges. Int. J. Med. Inform..

[10] Pham T. (2025). Ethical and Legal Considerations in Healthcare AI: Innovation and Policy for Safe and Fair Use. R. Soc. Open Sci..

[11] Bevilacqua R., Bailoni T., Maranesi E., Amabili G., Barbarossa F., Ponzano M., Virgolesi M., Rea T., Illario M., Piras E.M. (2025). Framing the Human-Centered Artificial Intelligence Concepts and Methods: Scoping Review. JMIR Hum. Factors.

[12] Li C. (2025). AI Alignment Is All Your Need for Future Drug Discovery. Front. Artif. Intell..

[13] Holzinger A., Saranti A., Angerschmid A., Retzlaff C.O., Gronauer A., Pejakovic V., Medel-Jimenez F., Krexner T., Gollob C., Stampfer K. (2022). Digital Transformation in Smart Farm and Forest Operations Needs Human-Centered AI: Challenges and Future Directions. Sensors.

[14] Chen Y., Clayton E.W., Novak L.L., Anders S., Malin B. (2023). Human-Centered Design to Address Biases in Artificial Intelligence. J. Med. Internet Res..

[15] Williams S.C., Zhou J., Muirhead W.R., Khan D.Z., Koh C.H., Ahmed R., Funnell J.P., Hanrahan J.G., Ali A.M., Ghosh S. (2024). Artificial Intelligence Assisted Surgical Scene Recognition: A Comparative Study Amongst Healthcare Professionals. Ann. Surg..

[16] Shulha M., Hovdebo J., D’Souza V., Thibault F., Harmouche R. (2024). Integrating Explainable Machine Learning in Clinical Decision Support Systems: Study Involving a Modified Design Thinking Approach. JMIR Form. Res..

[17] Sassi Z., Eickmann S., Roller R., Osmanodja B., Burchardt A., Tretter M., Samhammer D., Dabrock P., Möller S., Budde K. (2025). Human-centered AI in healthcare: Empowering patients and support persons in clinical decision-making. BMC Med. Inform. Decis. Mak..

[18] Behzadifar M., Azari S., Sajedimehr N., Aalipour A., Nematkhah M., Teli B.D., Martini M., Yarahmadi M., Behzadifar M. (2025). Challenges of Using Artificial Intelligence in Iran’s Health System: A Qualitative Study. J. Prev. Med. Hyg..

[19] Padela A.I., Hayek R., Tabassum A., Jotterand F., Qadir J. (2026). Assisting, Replicating, or Autonomously Acting? An Ethical Framework for Integrating AI Tools and Technologies in Healthcare. Bioethics.

[20] Coker E., Lockspeiser T., Hilgenberg S.L. (2026). Artificial Intelligence Healthcare Industry Expert Perspectives: How Artificial Intelligence Will Impact Physician Roles and Medical Education. Acad. Pediatr..

[21] Wei H., Trepanier S., Wei T., Marshall D., Wiley R.D., D’Agostino D. (2025). The Philosophy of Artificial Intelligence in Healthcare: Facilitating a Human-Centered Paradigm to Optimize Healthcare Outcomes. Healthc. Manag. Forum.

[22] Fritz R.L., Nguyen-Truong C.K.Y., May T., Wuestney K., Cook D.J. (2024). Bioethics Principles in Machine Learning-Healthcare Application Design: Achieving Health Justice and Health Equity. Harv. Public Health Rev..

[23] Henriksen A., Blond L. (2023). Executive-Centered AI? Designing Predictive Systems for the Public Sector. Soc. Stud. Sci..

[24] Trojánek M., Karvai J. (2025). Artificial Intelligence in Healthcare. Klin. Mikrobiol. Infekcni Lek..

[25] Armero W., Gray K.J., Fields K.G., Cole N.M., Bates D.W., Kovacheva V.P. (2022). A Survey of Pregnant Patients’ Perspectives on the Implementation of Artificial Intelligence in Clinical Care. J. Am. Med. Inform. Assoc..

[26] Rony M.K.K., Numan S.M., Akter K., Tushar H., Debnath M., Johra F.T., Akter F., Mondal S., Das M., Uddin M.J. (2024). Nurses’ Perspectives on Privacy and Ethical Concerns Regarding Artificial Intelligence Adoption in Healthcare. Heliyon.

[27] Hassan M., Borycki E.M., Kushniruk A.W. (2025). Artificial Intelligence Governance Framework for Healthcare. Healthc. Manag. Forum.

[28] Turchi T., Prencipe G., Malizia A., Filogna S., Latrofa F., Sgandurra G. (2024). Pathways to Democratic Healthcare: Envisioning Human-Centered AI-as-a-Service for Customized Diagnosis and Rehabilitation. Artif. Intell. Med..

[29] Holeman I., Kane D. (2019). Human-Centered Design for Global Health Equity. Inf. Technol. Dev..

[30] Isangula K., Pallangyo E.S., Ndirangu-Mugo E. (2024). Nurses’ and Clients’ Perspectives After Engagement in the Co-Designing of Solutions to Improve Provider-Client Relationships in Maternal and Child Healthcare: A Human-Centered Design Study in Rural Tanzania. BMC Nurs..

[31] Isangula K., Pallangyo E.S., Ndirangu-Mugo E. (2023). Interventions Co-Designed by Healthcare Providers and Clients for Improving Therapeutic Relationships in Maternal and Child Healthcare: A Pilot Study Using Human Centered Design in Rural Tanzania. BMC Nurs..

[32] Bangerter L.R., Looze M., Barry B., Harder K., Griffin J., Dezutter M., Khera N., Ailawadhi S., Schaepe K., Fischer K. (2022). A Hybrid Method of Healthcare Delivery Research and Human-Centered Design to Develop Technology-Enabled Support for Caregivers of Hematopoietic Stem Cell Transplant Recipients. Support. Care Cancer Off. J. Multinatl. Assoc. Support. Care Cancer.

[33] Mohammed S., Savage T., Smith J., Shepley M.M., White R.D. (2023). Reimagining the NICU: A Human-Centered Design Approach to Healthcare Innovation. J. Perinatol. Off. J. Calif. Perinat. Assoc..

[34] Chen E., Neta G., Roberts M.C. (2021). Complementary Approaches to Problem Solving in Healthcare and Public Health: Implementation Science and Human-Centered Design. Transl. Behav. Med..

[35] Matenga T.F.L., Herce M.E., Corbin J.H., Zulu J.M., Mwila C., Mandyata C., Frimpong C., Siame M., Shankalala P., Nachalwe N. (2026). Applying human-centered design to adapt a multifaceted implementation strategy for integrating HIV and NCD services in Lusaka, Zambia: Healthcare worker perspectives. PLoS Glob. Public Health.

[36] Fischer M., Safaeinili N., Haverfield M.C., Brown-Johnson C.G., Zionts D., Zulman D.M. (2021). Approach to Human-Centered, Evidence-Driven Adaptive Design (AHEAD) for Health Care Interventions: A Proposed Framework. J. General. Intern. Med..

[37] Salwei M.E., Davis S.E., Reale C., Novak L.L., Walsh C.G., Beebe R., Nelson S., Sundrani S., Rose S., Wright A. (2025). Human-centered design of an artificial intelligence monitoring system: The Vanderbilt Algorithmovigilance Monitoring and Operations System. JAMIA Open.

[38] Choudhury A., Shamszare H. (2026). Human Factors Influencing Trust in Healthcare Artificial Intelligence: Systematic Literature Review. IISE Trans. Occup. Ergon. Hum. Factors.

[39] Ackerhans S., Wehkamp K., Petzina R., Dumitrescu D., Schultz C. (2025). Perceived Trust and Professional Identity Threat in AI-Based Clinical Decision Support Systems: Scenario-Based Experimental Study on AI Process Design Features. JMIR Form. Res..

[40] Wysocki O., Davies J.K., Vigo M., Armstrong A.C., Landers D., Lee R., Freitas A. (2023). Assessing the Communication Gap Between AI Models and Healthcare Professionals: Explainability, Utility and Trust in AI-Driven Clinical Decision-Making. Artif. Intell..

[41] Wysocki O., Mak S., Frost H., Graham D.M., Landers D., Aslam T. (2025). Translating the Machine; an Assessment of Clinician Understanding of Ophthalmological Artificial Intelligence Outputs. Int. J. Med. Inform..

[42] Lopes S., Mascarenhas M., Fonseca J., Fernandes M.G.O., Leite-Moreira A.F. (2025). Unveiling the Algorithm: The Role of Explainable Artificial Intelligence in Modern Surgery. Healthcare.

[43] Ranwala R.A.D.L.M.K., Andrade A.Q. (2025). Enhancing AI Clinical Decision Support Trust: Design Workshop Insights from General Practitioners. Stud. Health Technol. Inform..

[44] Al-Bakri F.H., Bejuri W.M.Y.W., Al-Andoli M.N., Ikram R.R.R., Khor H.M., Sholva Y., Ariffin U.K., Yaacob N.M., Abas Z.A., Abidin Z.Z. (2025). A Feature-Augmented Explainable Artificial Intelligence Model for Diagnosing Alzheimer’s Disease from Multimodal Clinical and Neuroimaging Data. Diagnostics.

[45] Vehi J., Mujahid O., Beneyto A., Contreras I. (2025). Generative Artificial Intelligence in Diabetes Healthcare. iScience.

[46] Sümer Ö., Huber T., Duong D., Hanchard S.E.L., Conati C., André E., Solomon B.D., Waikel R.L. (2025). Evaluation of a Deep Learning and XAI Based Facial Phenotyping Tool for Genetic Syndromes: A Clinical User Study. medRxiv.

[47] Kelly A., Bhardwaj N., Sainte-Marie T.T.H., de Ven P.V., Melia R., Williams J.E., Mathiasen K., Nielsen A.S. (2025). Investigating How Clinicians Form Trust in an AI-Based Mental Health Model: Qualitative Case Study. JMIR Hum. Factors.

[48] Liu X., Chen Y., Guan W., Jiang P., Yan L., Fan M., Zhou Q. (2025). The Mediation of AI Trust on AI Uncertainties and AI Competence Among Nurses: A Cross-sectional Study. J. Adv. Nurs..

[49] Gotta J., Grünewald L.D., Koch V., Mahmoudi S., Bernatz S., Höhne E., Biciusca T., Gökduman A., Wolfram C., Booz C. (2025). Implementation of AI in Radiology: The Perspective of Referring Physicians. Insights Into Imaging.

[50] Ateş A.Y., Çetin G. (2026). Assessing Healthcare Professionals’ Attitudes Toward Artificial Intelligence in Turkey: A Cross-Sectional Study.

[51] N A., Chowdhury R.R., L P., Peter R.M., Vv A. (2024). Perception of the Adoption of Artificial Intelligence in Healthcare Practices Among Healthcare Professionals in a Tertiary Care Hospital: A Cross-Sectional Study. Cureus.

[52] Wang X., Fei F., Wei J., Huang M., Xiang F., Tu J., Wang Y., Gan J. (2024). Knowledge and attitudes toward artificial intelligence in nursing among various categories of professionals in China: A cross-sectional study. Front. Public Health.

[53] Azzam S., El-Morsy E.-M.A., Said A.S.A., Eissa N., Khalil D.M. (2025). Perception and Awareness of Healthcare Professionals Toward the Applications of Artificial Intelligence in Egyptian Healthcare Settings. Front. Artif. Intell..

[54] Oremule B., Saunders G.H., Kluk K., d’Elia A., Bruce I.A. (2024). Understanding, Experience and Attitudes Towards Artificial Intelligence Technologies for Clinical Decision Support in Hearing Health: A Mixed-Methods Survey of Healthcare Professionals in the UK. J. Laryngol. Otol..

[55] Petrica A., Marza A.M., Barsac C., Cebzan A., Dragan I., Zaharie D., Horhat R., Lungeanu D. (2025). Artificial Intelligence in Emergency Department Triage: Perspective of Human Professionals. Front. Digit. Health.

[56] Vo V., Chen G., Aquino Y.S.J., Carter S.M., Do Q.N., Woode M.E. (2023). Multi-Stakeholder Preferences for the Use of Artificial Intelligence in Healthcare: A Systematic Review and Thematic Analysis. Soc. Sci. Med..

[57] Tang J.S., Frazer H.M., Kunicki K., Basnayake P., Omori M., Lippey J. (2024). Australian Healthcare Workers’ Views on Artificial Intelligence in BreastScreen: Results of a Mixed Method Survey Study. Prev. Med. Rep..

[58] Roth J., Thunga S., Yoo J. (2025). Assessing the Landscape of AI-Powered Patient Documentation in Dermatology. J. Drugs Dermatol..

[59] Villringer K., Sokiranski R., Opfer R., Spies L., Hamann M., Bormann A., Brehmer M., Galinovic I., Fiebach J.B. (2025). An Artificial Intelligence Algorithm Integrated into the Clinical Workflow Can Ensure High Quality Acute Intracranial Hemorrhage CT Diagnostic. Clin. Neuroradiol..

[60] Eldesoky H.A.M., AlThubaity D., Shalby A.Y.M., Mohammed F.A. (2025). Solicitude Toward Artificial Intelligence Among Health Care Providers and Its Relation to Their Patient’s Safety Culture in Saudi Arabia. BMC Health Serv. Res..

[61] Dai Q., Li M., Yang M., Shi S., Wang Z., Liao J., Li Z., E W., Tao L., Tang Y.-D. (2025). Attitudes, Perceptions, and Factors Influencing the Adoption of AI in Health Care Among Medical Staff: Nationwide Cross-Sectional Survey Study. J. Med. Internet Res..

[62] Xiang Y., Zhao L., Liu Z., Wu X., Chen J., Long E., Lin D., Zhu Y., Chen C., Lin Z. (2020). Implementation of Artificial Intelligence in Medicine: Status Analysis and Development Suggestions. Artif. Intell. Med..

[63] Zheng L., Xiao Y. (2025). Refining AI Perspectives: Assessing the Impact of Ai Curricular on Medical Students’ Attitudes Towards Artificial Intelligence. BMC Med. Educ..

[64] Sadiq F., Gul R., Zuhra F., Khan M.K., Shah S.M.A., Uzma F., Khattak N.U.H., Alam W., Khan M.U. (2024). Knowledge, Attitude, and Practice (KAP) Regarding the Use of Artificial Intelligence in Hospital Settings in Mardan, Khyber Pakhtunkhwa, Pakistan. Cureus.

[65] Rony M.K.K., Parvin M.R., Wahiduzzaman M., Debnath M., Bala S.D., Kayesh I. (2024). I Wonder If My Years of Training and Expertise Will Be Devalued by Machines”: Concerns about the Replacement of Medical Professionals by Artificial Intelligence. SAGE Open Nurs..

[66] Banerjee S., Alsop P., Jones L., Cardinal R.N. (2022). Patient and Public Involvement to Build Trust in Artificial Intelligence: A Framework, Tools, and Case Studies. Patterns.

[67] Astobiza A.M., Alonso M., Lozano R.O. (2025). Trust and AI in Healthcare: A Systematic Review. Monash Bioeth. Rev..

[68] Kacafírková K.S., Poll A., Jacobs A., Cardone A., Ventura J.-J. (2025). Exploring Women’s Perceptions of Traditional Mammography and the Concept of AI-Driven Thermography to Improve the Breast Cancer Screening Journey: Mixed Methods Study. JMIR Cancer.

[69] Xu Y., Wan J., Wan B., Ding H. (2025). A Multi-Stakeholder Multicriteria Decision Analysis for Implantable Medical Devices Assessment in China. Front. Health Serv..

[70] Bruzzone S., Paoli G., Scillieri G.S., Sacile R., Giacomini M. (2025). Proposal of a Methodology to Enhance Mini-HTA Evaluations. Stud. Health Technol. Inform..

[71] Alluhaibi R., Alharbe N., Aljohani A., Mamlook R.E.A. (2023). Selection of an Efficient Classification Algorithm for Ambient Assisted Living: Supportive Care for Elderly People. Healthcare.

[72] Sriharan A., Kuhlmann E., Correia T., Tahzib F., Czabanowska K., Ungureanu M.-I., Kumar B.N. (2025). Artificial Intelligence in Healthcare: Balancing Technological Innovation with Health and Care Workforce Priorities. Int. J. Health Plan. Manag..

[73] Tchaicha S., Auvin S., Beniczky S., Brunklaus A., Lagae L., Perucca E., Surges R., Adler S., Helmstaedter C., Jansen F. (2026). Areas of Research Priorities in Epilepsy: A Position Paper of the European Reference Network for Rare and Complex Epilepsies, EpiCARE. Epilepsia Open.

[74] Voigtlaender S., Pawelczyk J., Geiger M., Vaios E.J., Karschnia P., Cudkowicz M., Dietrich J., Haraldsen I.R.J.H., Feigin V., Owolabi M. (2024). Artificial Intelligence in Neurology: Opportunities, Challenges, and Policy Implications. J. Neurol..

[75] Byrne M.D. (2021). Reducing Bias in Healthcare Artificial Intelligence. J. Perianesthesia Nurs. Off. J. Am. Soc. Perianesthesia Nurses.

[76] Liu Y., Deng K., Zhang C., Yuan Z., Zhou J., Liu C. (2026). The Rise of Intelligent Plastic Surgery: A 10-Year Bibliometric Journey Through AI Applications, Challenges, and Transformative Potential. Aesthetic Plast. Surg..

[77] Ralevski A., Taiyab N., Nossal M., Mico L., Piekos S.N., Hadlock J. (2024). Using Large Language Models to Annotate Complex Cases of Social Determinants of Health in Longitudinal Clinical Records. medRxiv.

[78] Choe M., Shim J.H., Heo C.Y. (2022). A Comparative Study of Regulatory Perspectives on Innovative Medical Devices in Korea and the United States. Expert Rev. Med. Devices.

[79] Ducret M., Mörch C.-M., Karteva T., Fisher J., Schwendicke F. (2022). Artificial Intelligence for Sustainable Oral Healthcare. J. Dent..

[80] Nankya H., Mathews D., Ferryman K., Kane O., Ali J. (2025). Community Engagement for Artificial Intelligence Health Research in Africa. Wellcome Open Res..

[81] Leenen J.P.L., Hiemstra P., Hoeve M.M.T., Jansen A.C.J., van Dijk J.D., Vendel B., Versteeg G., Hakvoort G.A., Hettinga M. (2025). Exploring the Complex Nature of Implementation of Artificial Intelligence in Clinical Practice: An Interview Study with Healthcare Professionals, Researchers and Policy and Governance Experts. PLoS Digit. Health.

[82] Fazakarley C.A., Breen M., Leeson P., Thompson B., Williamson V. (2023). Experiences of Using Artificial Intelligence in Healthcare: A Qualitative Study of UK Clinician and Key Stakeholder Perspectives. BMJ Open.

[83] de Graaf Y., Ahmed A., Sanges C., Herbst L., Vrijhoef H.J.M. (2025). Societal Factors Influencing the Implementation of AI-Driven Technologies in (Smart) Hospitals. PLoS ONE.

[84] Newlands R., Bruhn H., Díaz M.R., Lip G., Anderson L.A., Ramsay C. (2024). A Stakeholder Analysis to Prepare for Real-World Evaluation of Integrating Artificial Intelligent Algorithms into Breast Screening (PREP-AIR Study): A Qualitative Study Using the WHO Guide. BMC Health Serv. Res..

[85] Yu J.Y., Hong S., Lee Y.C., Lee K.H., Lee I., Seo Y., Kang M., Kim K., Cha W.C., Shin S.-Y. (2022). Stakeholders’ Requirements for Artificial Intelligence for Healthcare in Korea. Healthc. Inform. Res..

[86] Cleofas J.V. (2026). Towards a Framework for Human Rights-Based Stakeholdership of Nursing Academia in the Healthcare Artificial Intelligence Governance Ecosystem: A Discussion Paper. Nurse Educ. Pract..

[87] Loftus T.J., Balch J.A., Abbott K.L., Hu D., Ruppert M.M., Shickel B., Ozrazgat-Baslanti T., Efron P.A., Tighe P.J., Hogan W.R. (2024). Community-Engaged Artificial Intelligence Research: A Scoping Review. PLoS Digit. Health.

[88] Janssen A., Shah K., Teede H., Shaw T. (2025). Implementers Perspectives on the Routine Use of Artificial Intelligence in Health Services: A Qualitative Study Using the Consolidated Framework for Implementation Research (CFIR). Health Serv. Insights.

[89] Bayer M., Eisawi A. (2025). Exploring the Landscape of Artificial Intelligence in Saudi Arabia’s Healthcare Sector: Current Trends and Challenges. Cureus.

[90] Goktas P., Grzybowski A. (2025). Shaping the Future of Healthcare: Ethical Clinical Challenges and Pathways to Trustworthy AI. J. Clin. Med..

[91] Evans T.D., Ahmad O., Alderman J.E., Bailey G., Bannister P., Barlow N., Davison N., Isaac A., Kale A.U., MacDonald T. (2025). The Role of Procurement Frameworks in Responsible AI Innovation in the National Health Service: A Multi-Stakeholder Perspective. Front. Health Serv..

[92] Pavuluri S., Sangal R., Sather J., Taylor R.A. (2024). Balancing Act: The Complex Role of Artificial Intelligence in Addressing Burnout and Healthcare Workforce Dynamics. BMJ Health Care Inform..

[93] Ejaz H., McGrath H., Wong B.L., Guise A., Vercauteren T., Shapey J. (2022). Artificial Intelligence and Medical Education: A Global Mixed-Methods Study of Medical Students’ Perspectives. Digit. Health.

[94] Kedar S., Khazanchi D. (2023). Neurology Education in the Era of Artificial Intelligence. Curr. Opin. Neurol..

[95] Reuben J.S., Meiri H., Arien-Zakay H. (2024). AI’s Pivotal Impact on Redefining Stakeholder Roles and Their Interactions in Medical Education and Health Care. Front. Digit. Health.

[96] Yang Y., Cui Y.U., Wang Y.T., Xue P., Zhai X.M., Qiao Y.L. (2025). Interpretation of the WHO’s “Ethics and Governance of Artificial Intelligence for Health: Guidance on Large Multi-Modal Models”and its implications for China. Zhonghua Yu Fang Yi Xue Za Zhi [Chin. J. Prev. Med.].

[97] Jung J., Lee H., Jung H., Kim H. (2023). Essential Properties and Explanation Effectiveness of Explainable Artificial Intelligence in Healthcare: A Systematic Review. Heliyon.

[98] Kim J.Y., Hasan A., Kellogg K.C., Ratliff W., Murray S.G., Suresh H., Valladares A., Shaw K., Tobey D., Vidal D.E. (2024). Development and preliminary testing of Health Equity Across the AI Lifecycle (HEAAL): A framework for healthcare delivery organizations to mitigate the risk of AI solutions worsening health inequities. PLoS Digit. Health.

[99] Zhou Z., Xiang Y., Liu C., Huang X., Fu Y. (2025). Quantitative Evaluation and Optimization of AI Policy and Regulatory Texts for Smart Healthcare. Front. Public Health.

[100] Ahmad A., Vallès Y., Idaghdour Y. (2025). Bias in AI Systems: Integrating Formal and Socio-Technical Approaches. Front. Big Data.

[101] Tripathi S., Augustin A., Dako F., Kim E. (2022). Turing Test-Inspired Method for Analysis of Biases Prevalent in Artificial Intelligence-Based Medical Imaging. AI Ethics.

[102] Hopkins J.J., Keane P.A., Balaskas K. (2020). Delivering Personalized Medicine in Retinal Care: From Artificial Intelligence Algorithms to Clinical Application. Curr. Opin. Ophthalmol..

[103] Barai P., Leroy G., Bisht P., Rothman J.M., Lee S., Andrews J., Rice S.A., Ahmed A. (2024). Crowdsourcing with Enhanced Data Quality Assurance: An Efficient Approach to Mitigate Resource Scarcity Challenges in Training Large Language Models for Healthcare. AMIA Jt. Summits Transl. Sci. Proc..

[104] Lee C., Kumar S., Vogt K.A., Munshi M., Tallapudi P., Vogt A., Awad H., Khan W. (2025). Democratizing Cost-Effective, Agentic Artificial Intelligence to Multilingual Medical Summarization Through Knowledge Distillation. Sci. Rep..

[105] Chang W.-H., Chang M.-C., Chang M.-H., Chan Y.-K., Hsieh M.Y. (2025). Digital Health Transformation: An Empirical Analysis of Sustainable Healthcare Through the Various Applications of Artificial Intelligence Internet of Things Technology. Digit. Health.

[106] Doo F.X., Parekh V.S., Kanhere A., Savani D., Tejani A.S., Sapkota A., Yi P.H. (2024). Evaluation of Climate-Aware Metrics Tools for Radiology Informatics and Artificial Intelligence: Toward a Potential Radiology Ecolabel. J. Am. Coll. Radiol..

[107] Rogers W.A., Draper H., Carter S.M. (2021). Evaluation of Artificial Intelligence Clinical Applications: Detailed Case Analyses Show Value of Healthcare Ethics Approach in Identifying Patient Care Issues. Bioethics.

